# Intersectionality and mental health in university students: a jeopardy index approach

**DOI:** 10.11606/s1518-8787.2025059006197

**Published:** 2025-03-24

**Authors:** Juliana Dias de Lima, Jessica Plácido, Beatriz Andrade, Letícia Dalcero Abend, Aline Josiane Waclawovsky, Daniel Alves Pires, Danilo Rodrigues Pereira da Silva, Fabianna Resende de Jesus-Moraleida, Helena Ferreira Moura, Nicole Leite Galvão Coelho, Renato Sobral Monteiro-Junior, Thiago Sousa Matias, Felipe Barreto Schuch, Andrea Camaz Deslandes

**Affiliations:** I Universidade Federal do Rio de Janeiro. Instituto de Psiquiatria. Rio de Janeiro, RJ, Brasil; II Universidade Federal de Santa Maria. Departamento de Métodos e Técnicas Desportivas. Santa Maria, RS, Brasil; III Universidade Federal do Pará. Programa de Pós-Graduação em Ciências do Movimento Humano. Castanhal, PA, Brasil; IV Universidade Federal de Sergipe. Departamento de Educação Física. São Cristóvão, SE, Brasil; V Universidad Autónoma de Chile. Ciencias de la Salud. Providencia, Chile; VI Universidade Federal do Ceará. Departamento de Fisioterapia. Programa de Pós-Graduação em Fisioterapia e Funcionalidade. Fortaleza, CE, Brasil; VII Universidade de Brasília. Faculdade de Medicina. Brasília, DF, Brasil; VIII Universidade Federal do Rio Grande do Norte. Departamento de Fisiologia e Comportamento. Natal, RN, Brasil; IX Universidade Estadual de Montes Claros. Departamento de Educação Física. Montes Claros, MG, Brasil; X Universidade Federal de Santa Catarina. Departamento de Educação Física. Florianópolis, SC, Brasil

**Keywords:** Mental Health, Mental Disorders, Ethnic and Racial Minorities, Sexual and Gender Minorities, Health Status Disparities

## Abstract

To explore the associations between current mental health symptoms and social disparities in university students.

We recruited participants from nine public universities in Brazil, from August to November 2022, using online advertisements and in-person lectures. All participants completed an online survey containing social (sex, race/color, gender identity, sexual orientation, and income) and mental health assessments. The Jeopardy index was composed of social variables. The index considered zero points for subjects with less oppressive experienced characteristics (men, White, cisgender, heterosexual, higher income) and one point for the opposite characteristics. We defined six clusters according to Jeopardy Index results: 0, 1, 2, 3, 4, and 5 points, with the greatest number of points representing the most disadvantaged group. The mental health symptoms were assessed on two levels. First by the “DSM-5 Self-Rated Level 1 Cross-Cutting Symptom Measure – Adult,” and second by the “Patient Health Questionnaire-9,” and the “Generalized Anxiety Disorder Questionnaire-7.” Adjusted Odds Ratio (OR) analyses was performed for age and educational level.

748 participants were allocated into the six Jeopardy clusters: 0 (n = 46; 6.1%), 1 (n = 112; 15.0%), 2 (n = 163; 21.8%), 3 (n = 218; 29.1%), 4 (n = 171; 22.9%), and 5 (n = 38; 5.1%). It was observed a high prevalence of anxiety (42.5%) and depression (51.0%), however, the less privileged group (5) had a higher risk of having severe symptoms of anxiety (OR = 6.21; 1.51–25.58; p < 0.01) and depression (OR = 8.60; 2.15–34.43; p < 0.01), compared against the most privileged group.

Although anxiety and depressive symptoms were highly prevalent for all participants, these disorders are not equally distributed in this population and the intersectionality between social factors plays an important role in contributing to these differences.

## INTRODUCTION

Among mental disorders, anxiety and depression emerged as the two most widespread conditions, impacting around 580 million individuals worldwide^1^. In addition to their high prevalence, mental disorders pose a public health challenge globally, ranking among the top ten causes of global burden^1^. Thus, suicide stands out as the fourth leading cause of death among young people aged 15 to 29 years old^2^.

In late adolescence and emerging adulthood, there is a peak onset of several common mental and substance use disorders^3^. Coinciding with this stage of life, many young adults enroll in college, leading to an anticipated high prevalence of mental disorders among the university student population^4^. Moreover, the transition to university is marked by numerous changes and newfound responsibilities, and financial and/or academic demands, potentially contributing to social instability and heightened distress^5^. Further, the changes in lifestyle behaviors can be important risk factors to mental disorders during this period^5 , 6^

Evidence suggests that approximately one-third of first-year university students (30%) experience a mental health or substance use problem within their initial year at the institution^4^. Even though most research on university students’ mental health focuses on high-income countries^7^, a comprehensive survey conducted by the World Health Organization across six high-income countries and two upper-middle-income countries^4^ revealed a widespread distribution of mental health problems among the student population. However, social inequalities and poor mental health are known to be inseparable, and that social disparities negatively affects a person’s access to treatment globally^8^.

Access to mental health care is influenced by various micro and macro-level factors^9^. At the macro-level perspective, there is an pervasive impact of economic and political forces of the country, such as the healthcare policies, the income level, and cultural barriers, including social stigma^9^. A recent Lancet Commission on global mental health underscored that sustainable efforts in mental health constitute a global public good^10^.

Furthermore, the Commission emphasized that mental health problems exist on a continuum of progressively severe conditions, in which individuals with mental disorders have complex needs, resultant from a unique interplay of social and biological influences throughout one’s lifetime^10^. These influences could be observed as micro-level factors associated with subjective mental health. Accordingly, the commission underscored the need to prioritize vulnerable populations subjected to discrimination based on social factors such as sex, race, ethnicity, and sexual orientation^9 , 10^. Consequently, the Commission advocates for a focus on intersectional social factors to identify specific risk protection actions in mental health.

Intersectionality is a term coined by Kimberly Crenshaw in the 1990s to elucidate the exclusion of Black women from discussions within White Feminism^11^. This perspective posits that various social factors, including race, sex, gender identity, sexual orientation, and socioeconomic status, intersect at the individual (micro) and at society (macro) level, which encompass systems of privileges and oppression, and issues such as sexism, racism, and heterosexism^11^. Fundamental to the concept of intersectionality is the recognition that these social factors are not unidimensional and independent, instead, they are multiple, interrelated, and mutually constitutive^12^.

The US Department of Health and Human Services report on health disparities suggests that while one single social or sociodemographic factor can be employed to understand or address health disparities, this approach overlooks the nuanced interactions among multiple social factors^13^. Furthermore, it neglects the social discrimination that arises from the interplay of these intersecting categories, resulting in disparities and social inequalities in health^12 , 13^.

Among university students, it is known that the new challenges and responsibilities of the academic lifespan pose risks for elevated stress and mental health problems^14^. Regarding financial problems, the educational debt system seems to be a stressor associated with decreased mental health, especially for Black and Latino students when compared with their White counterparts^15^. Besides socioeconomic problems, interpersonal factors for racism-related stress, perceived stigma, and discrimination are also associated with poor mental health in Black and Latino students^16^. Sexual minority students also deal with perceived stigma, as well as fear of violence and lack of a sense of belonging. They had higher risks for mental distress and depression, self-injuries, and suicidal thoughts and behaviors^17^.

Despite several studies investigating the mental health of social minorities in universities, there is a gap in understanding the interplay of multiple social factors in mental health, particularly in Brazil, which is a country with approximately 203 million people, most of whom are Non-White (56.5%)^18^. Among these vulnerable population, Black and Mixed-race people face lower income, reduced educational opportunities, poorer living conditions, and higher rates of death by homicide^19^. Given the shortage of studies concerning mental health and intersectionality in Brazilian university students, this paper aims to explore the associations between current mental health symptoms and social disparities in university students.

## METHODS

### Study Design, Setting, and Participants

In this multicenter cross-sectional study, university students enrolled in both undergraduate and postgraduate programs were recruited from nine Brazilian public universities representing the country’s diverse regions, from South, Southeast, North, Northeast, and Midwest. The institutions enrolled in this study were: Universidade Federal de Santa Maria (UFSM), Universidade Federal de Santa Catarina (UFSC), Universidade Federal do Rio de Janeiro (UFRJ), Universidade Estadual de Montes Claros (UNIMONTES), Universidade Federal do Pará (UFPA), Universidade Federal do Rio Grande do Norte (UFRN), Universidade Federal do Ceará (UFC), Universidade Federal de Sergipe (UFS), and Universidade de Brasília (UNB). It was a convenience sample conducted from August to November 2022. The students were recruited online and in in-person lectures, in accordance with relevant local ethics and data privacy laws and policies. Inclusion criteria were those aged > 18 years old and enrolled in the university. The participants agreed to be part of this research by virtually signing the informed consent form and answering the online questionnaire in the REDCap platform. The Research Ethics Committee of the IPUB-UFRJ approved this study under registration permit CAAE:55481422.5.1001.5346.

The questionnaire comprised assessments of sociodemographic data and mental health problems, of which the latter was conducted at two levels. Initially, the participants completed the “DSM-5 Self-Rated Level 1 Cross-Cutting Symptom Measure (CCSM-1),” a self-reported questionnaire assessing crucial domains across various psychiatric diagnoses. The instrument screens for symptoms of anxiety, depression, suicidal ideation, anger, mania, somatic symptoms, psychosis, dissociation, personality functioning, memory and sleep problems, repetitive thoughts and behaviors, and substance use. It employs a 5-point scale (scoring from 0 to 4) to gauge the frequency with which an individual has been troubled by any of the aforementioned symptoms over the past two weeks. Respondents who received a positive screening at the level one instrument (rating ≥ 2) for depression and anxiety were directed to a level two instrument for a more in-depth investigation of these symptoms.

Depressive symptoms were assessed using the “Patient Health Questionnaire-9 (PHQ-9),”which comprises nine items to evaluate the presence of symptoms over the last two weeks. The scoring ranges from 0 to 27, with higher scores indicating greater symptom severity (5–9 = mild; 10–14 = moderate; 15–19 = moderate to severe; 20–27 = severe). To assess anxiety symptoms, the “Generalized Anxiety Disorder Questionnaire-7 (GAD-7)” was employed, consisting of seven items with the score ranging from 0 to 21. Similar to the PHQ-9, the GAD-7 evaluates symptoms over the two preceding weeks, and higher scores indicate greater symptom severity (5–9 = mild; 10–14 = moderate; ≥ 15 = severe). For both questionnaires, participants with scores ≥ 10 points were classified as cases of depression or anxiety. These instruments have been translated and validated for the Brazilian population^20 - 22^.

### Procedures and Statistical Analysis

**Figure 1.**
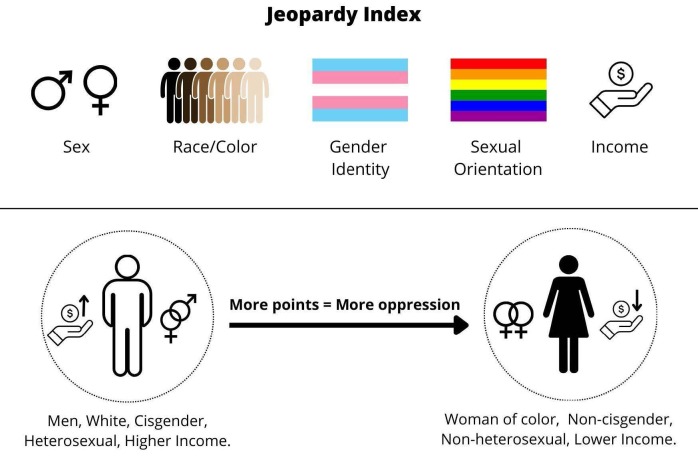
Representation of the Jeopardy Index approach and the sociodemographic variables references.

To categorize our data into clusters, we based the sociodemographic classification on the multiple jeopardy, which is a concept rooted in the principle of intersectionality. The multiple jeopardy theory posits that disadvantaged identities, regarding gender or race/color for example, contribute to a complex risk of oppression and social discrimination in an individual’s experience. This effect is exacerbated by the interdependent combination of identities, resulting in a multiplicative compound rather than a simple cumulative idea^23^. Consequently, we classified the sample using the Jeopardy Index composite score (Figure 1). The index assigns zero points to subjects with less oppressed characteristics (men, White, cisgender, heterosexual, higher income) and one point to those with the opposite characteristics (woman, non-White, non-heterosexual, lower income)^24^. Income was categorized into three brackets, based on the 2022 Brazilian minimum wage BRL (R$ 1.287,00). Income was classified into: < 2 minimum salaries, from 2 to 6 salaries, and > 6 minimum salaries. For the index calculation, it was assigned zero points to the higher income category and two points the lower income category. The Jeopardy Index score ranged from zero to six points, which were classified into the six clusters (0, 1, 2, 3, 4, and 5 points) for statistical analysis.

A descriptive analysis of the sociodemographic data was undertaken. The Chi-square test was employed to evaluate differences between groups, including sociodemographic and mental health problems. A sub-analysis of symptom severity distributed by each Jeopardy Index cluster was undertaken, only with those respondents who screened positive in the Level 1 assessment. Binary and multinomial logistic regression models were used to estimate the odds ratio (OR) with 95% confidence intervals (95%CI) for depression and anxiety within each Jeopardy Index group category. An independent model for each outcome was performed: binary analyses were performed to assess the presence of depression and anxiety cases; and multinomial analyses were conducted to verify symptoms severity. The reference group used in logistic regression analyses was the most privileged one, according to the Jeopardy Index classification (group 0). Adjusted odds ratio (Adj. OR) were conducted for age and educational level, since the prevalence of anxiety and depression are apparently higher in postgraduate students^25^. All statistical analyses were carried out using SPSS® version 26.0, with a significance level set at p ≤ 0.05.

## RESULTS

In this study, 868 university students were screened at first, of which120 were excluded, since they interrupted the survey after answering only the sociodemographic section. Thus, the analytical sample consists of 748 participants, mostly female (n = 416; 61.6%), identifying as Black and Mixed-race (n = 414; 55.3%), cisgender (n = 727; 97.1%), and heterosexual (n = 556; 74.3%), with a mean age of 23.2 years (SD = 6.1). Most participants reported a lower family income (R$), receiving less than two Brazilian minimum wages (n = 350; 46.7%), followed by two to six wages (n = 236; 31.5%), and more than six wages (n = 151; 20.1%). The distribution of participants by region was as follows: South (n = 119; 15.5%), Southeast (n = 173; 23.1%), North (n = 133; 17.8%), Northeast (n = 247; 33.0%), and Midwest (n = 60; 8.0%); in addition to other16 participants who did not respond to the university site question (2.1%).

**Figure 2.**
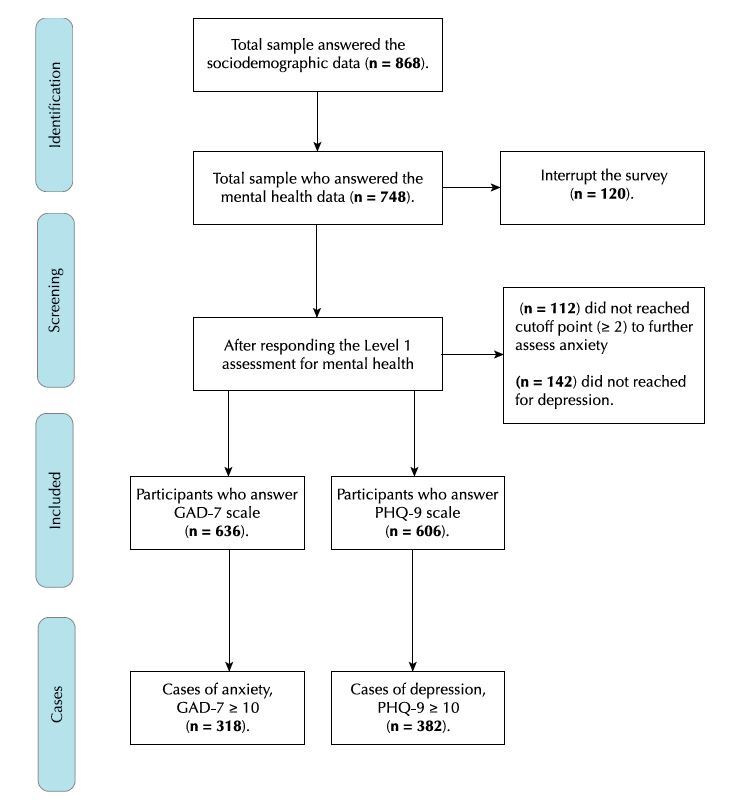
Flowchart of the survey collection data process.

The process of mental health data collection occurred as follows: all participants answered the first part of the screening section regarding mental health problems using the CCSM-1 scale. Of them (n = 748), n = 636 participants reached the cutoff point (≥ 2) in the level one assessment for anxiety, and n = 606 reached the cutoff point for depression. Consequently, these participants were further investigated for these symptoms, answering the specific assessment for anxiety (GAD-7 scale) and depression (PHQ-9 scale). A flowchart of the survey process of data collection is presented in Figure 2.

The prevalence of anxiety and depression cases in the study sample was 42.5% (n = 318) and 51.0% (n = 382), respectively. According to the GAD-7 scale, the prevalence of anxiety, categorized by symptom severity, was as follows: mild (n = 230; 30.8%), moderate (n = 166, 22.2%), and severe (n = 152; 20.3%). Meanwhile, 26.7% (n = 200) of the students reported no current symptoms of anxiety. Regarding the severity categories of depressive symptoms as per the PHQ-9, prevalence was observed across the following four classifications: mild (n = 183; 24.5%), moderate (n = 167; 22.3%), moderate to severe (n = 119; 15.9%), and severe (n = 96; 12.8%). Additionally, 24.5% (n = 183) of the students did not report any depressive symptoms.

**Figure 3.**
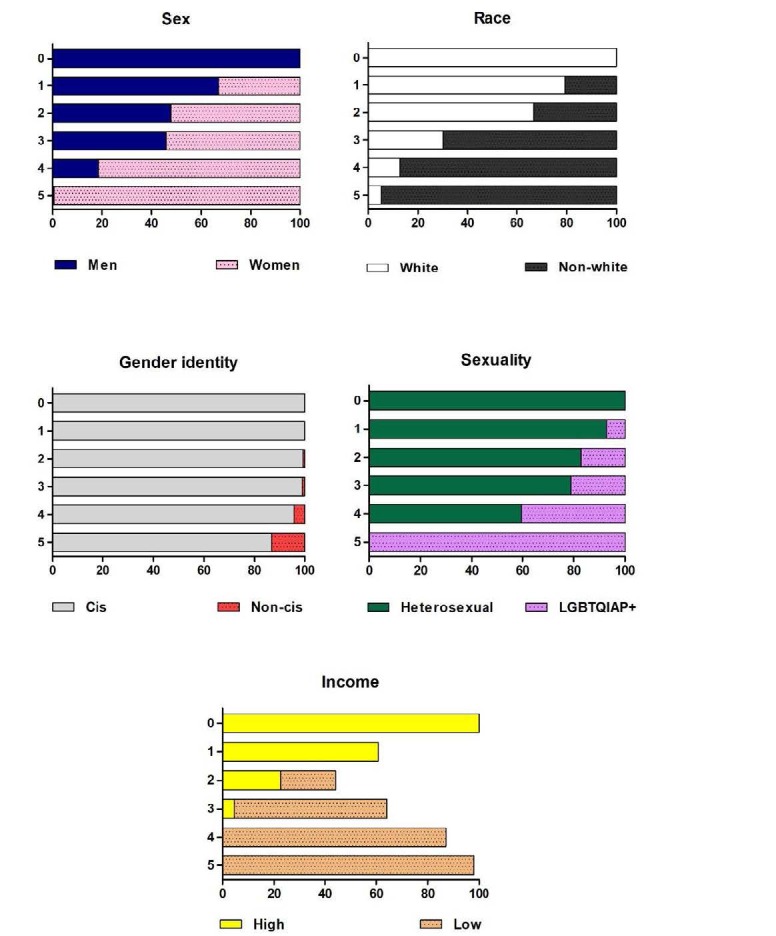
Prevalence of sociodemographic factors by the Jeopardy Index classification.

To elucidate socio-characteristics of the sample, Table 1 presents the prevalence and association between sociodemographic data, mental health, and each Jeopardy Index cluster group. Additionally, Figure 3 illustrates the prevalence of each dichotomy Jeopardy Index variable. The Jeopardy Index classification ranked the sample (n = 748) into six cluster groups, resulting in: 46 students classified in group 0 (6.1%), 112 in group 1 (15.0%), 163 in group 2 (21.8%), 218 in group 3 (29.1%), 171 in group 4 (22.9%), and 38 students in group 5 (5.1%).

**Table 1.** Association between sociodemographic characteristics, mental health problems, and the Jeopardy Index clusters.

**Variable**	**Jeopardy Index Groups N (%)**						**P-value**
	**0**	**1**	**2**	**3**	**4**	**5**	
**Total**	**46**	**112**	**163**	**218**	**171**	**38**	
**Sex**							**<0.001**
Male	46 (100)	75 (67.0)	78 (47.9)	100 (45.9)	32 (18.7)	1 (0.6)	
Female	0 (0.0)	37 (33.0)	85 (52.1)	118 (54.1)	139 (81.3)	37 (99.4)	
**Race/color**							**<0.001**
White	46 (100)	89 (79.4)	109 (66.9)	66 (30.2)	22 (12.8)	2 (5.3)	
Non-White	0 (0.0)	23 (20.6)	54 (33.1)	152 (69.8)	149 (87.2)	36 (94.7)	
**Gender**							**<0.001**
Cisgender	46 (100)	112 (100)	162 (99.4)	216 (99.2)	164 (95.9)	33 (86.8)	
Non-Cisgender	0 (0.0)	0 (0.0)	1 (0.6)	2 (0.8)	7 (4.1)	5 (13.2)	
**Sexual Orientation**							**<0.001**
Heterosexual	46 (100)	104 (92.8)	135 (82.9)	174 (78.9)	102 (59.6)	0 (0.0)	
Non-Heterosexual	0 (0.0)	8 (7.2)	28 (17.1)	44 (21.1)	69 (40.4)	38 (100)	
**Income**							**<0.001**
< 2	0 (0.0)	0 (0.0)	35 (21.5)	130 (59.6)	149 (87.1)	37 (97.9)	
2–6	0 (0.0)	44 (39.3)	91 (55.8)	78 (35.8)	22 (12.9)	1 (2.1)	
> 6	46 (100)	68 (60.7)	37 (22.7)	10 (4.6)	0 (0.0)	0 (0.0)	
**Mental Health Prevalences**							
**Anxiety**							**<0.001**
GAD-7 (≥ 10)	17 (37.0) ^#∆^	36 (32.1) ^#∆^	62 (38.0) ^#∆^	86 (39.4) ^#∆^	93 (54.3)	24 (63.1)	
**Severity**							**<0.001**
Mild	14 (30.4)	44 (39.3)	50 (30.6)	72 (33.0)	42 (24.5)	8 (21.0)	
Moderate	13 (28.6)	23 (19.0)	39 (23.9)	43 (19.7)	38 (22.2)	10 (26.3)	
Severe	4 (8.7)	13 (11.6)	23 (14.1)	43 (19.7)	55 (32.1)	14 (36.8)	
**Depression**							**<0.001**
PHQ-9 (≥ 10)	18 (39.1) ^#∆^ °	44 (39.3) ^#∆^ °	75 (46.0) ^#∆^ °	114 (52.3) ^∆^	102 (59.6)	29 (76.3)	
**Severity**							**<0.001**
Mild	17 (37.0)	33 (29.5)	46 (28.2)	44 (20.1)	37 (21.6)	6 (15.8)	
Moderate	9 (19.5)	23 (20.5)	34 (20.8)	56 (25.6)	38 (22.2)	7 (18.4)	
Moderate to severe	4 (8.7)	15 (13.4)	30 (18.4)	33 (15.1)	31 (18.1)	7 (18.4)	
Severe	5 (10.9)	6 (5.3)	11 (6.7)	26 (12.0)	33 (19.2)	15 (39.4)	

Note: Income values are reported in Brazilian Real (R$) minimum salaries. Converted to U.S Dollar ($), the values are: < 477.33; 407.54–1,756.17; > 1,756.37. The prevalence of anxiety and depression cases are reported based on the cutoff points of GAD-7 and PHQ-9 scales. Of all participants, n = 636 were further assessed for anxiety, and n = 606 for depression. ° = Significant difference compared to Group 3; # = Significant difference compared to Group 4; ∆ = Significant difference compared to Group 5.

**Figure 4.**
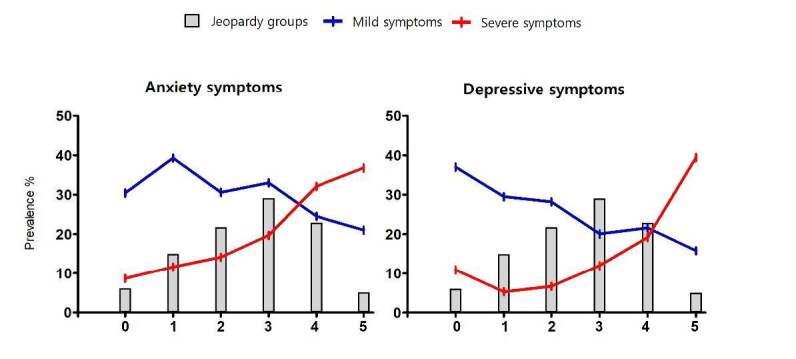
Distribution of Jeopardy Index and prevalence of anxiety and depressive symptom severity according to the Jeopardy Index groups.

Regarding the prevalence of anxiety cases, Chi-square analyses revealed significant differences between the first four cluster groups (0, 1, 2, and 3) and the last two groups (4 and 5) of the Jeopardy Index classification (Table 1). Additionally, odds ratio analyses showed that these last two groups (4 and 5) also had significantly higher risks of experiencing anxiety when compared with the other cluster groups (Table 2). The Chi-square analyses for the prevalence of depression cases showed very similar results to those for anxiety, with significant differences observed between the first cluster groups (0, 1, and 2) and the last ones (3, 4, and 5) (Table 1). Furthermore, the odds ratio analyses revealed significantly higher chances of experiencing depression from the third group of the Jeopardy Index on. This suggests that accumulating just three points of intersectionality already elevates the odds of depressive cases (Table 2). The prevalence of mild and severe symptoms in each Jeopardy group is highlighted in Figure 4.

Chi-square analyses showed no differences between groups 4 and 5 regarding the prevalence of clinical cases of anxiety and depression (≥ 10 points on the clinical scales). However, those final cluster groups (4 and 5) exhibit substantial similarity in the sociodemographic characteristics allied with the Jeopardy Index approach. Both groups predominantly comprised women (81.3%; 99.4%), people of color (87.2%; 94.7%), and lower-income individuals (100%), with a smaller proportion identifying as non-cisgender (4.1%; 13.2%). The sole distinguishing factor observed in group 5 was sexuality, since all participants (100%) identified as non-heterosexual (refer to Figure 3).

Figure 4 demonstrates the changes in the distribution of symptom severity based on intersectionality factors, revealing a crescent pattern of severe mental health problems symptoms with the cumulative effect of social oppressive characteristics. To comprehend the risks associated with mental health problems according to the Jeopardy classification, a sub analysis with adjusted logistic regression models was conducted only with the participants who answered the GAD-7 (n = 636) and PHQ-9 scale (n = 606) (Table 2). Non-adjusted odds ratio is presented in Table 3.

In terms of anxiety symptom severity, our observations indicate that university students who accumulate more than four or five points of intersectionality (groups 4 and 5) show a significant increase in odds of experiencing severe anxiety symptoms by almost five and six times, respectively. Conversely, for depressive symptoms, accumulating three points in the Jeopardy Index already poses a significant risk for moderate to severe symptoms, with the odds gradually increasing with the addition of one or two social factors. Individuals who accumulate five points in the Jeopardy Index (group 5) present alarming odds ratio, being nearly nine times more likely to have severe depressive symptoms, suggesting a substantially higher likelihood of struggling with depression.

**Table 2.** Odds ratio analysis of symptom severity distribution according to each Jeopardy Index group in Brazilian university students.

**Mental Health**	**Adj. OR**	**CI 95%**	P-value
**Anxiety symptoms GAD-7 (≥** **10)**			
1	0.77	(0.35–1.68)	0.52
2	1.02	(0.49–2.12)	0.95
3	1.03	(0.50–2.10)	0.92
4	2.14	(1.03–4.46)	**0.04**
5	2.57	(0.98–6.76)	**0.05**
**Moderate symptoms**			
1	0.60	(0.24–1.52)	0.29
2	0.91	(0.38–2.18)	0.84
3	0.66	(0.28–1.54)	0.33
4	0.99	(0.41–2.38)	0.98
5	1.36	(0.41–4.53)	0.60
**Severe symptoms**			
1	1.21	(0.33–4.35)	0.76
2	1.80	(0.53–6.10)	0.34
3	2.06	(0.63–6.72)	0.22
4	4.78	(1.46–15.65)	**0.01**
5	6.21	(1.51–25.58)	**0.01**
**Depressive symptoms PHQ-9 (≥** **10)**			
1	1.52	(0.75–3.28)	0.28
2	1.66	(0.81–3.41)	0.16
3	2.58	(1.27–5.25)	**<0.01**
4	3.04	(1.47–6.28)	**<0.01**
5	5.92	(2.00–17.50)	**<0.01**
**Moderate symptoms**			
1	1.42	(0.53–3.78)	0.48
2	1.49	(0.59–3.78)	0.39
3	2.48	(1.00–6.15)	**0.04**
4	2.05	(0.80–5.21)	0.13
5	2.23	(0.57–8.70)	0.24
**Moderate to severe**			
1	2.23	(0.63–7.85)	0.21
2	3.10	(0.94–10.20)	0.06
3	3.32	(1.01–10.91)	**0.04**
4	3.89	(1.17–12.87)	**0.02**
5	5.23	(1.11–24.63)	**0.03**
**Severe symptoms**			
1	0.72	(0.19–2.73)	0.63
2	0.88	(0.26–2.92)	0.83
3	2.08	(0.68–6.39)	0.19
4	3.26	(1.07–9.90)	**0.03**
5	8.60	(2.15–34.43)	**<0.01**

Note: Note: Adj. OR = Adjusted Odds Ratio by age and education level. The reference groups used for the analyses of symptoms severity were “mild severity” and the “zero points of Jeopardy Index.” The number of participants further assessed for anxiety and depression were, respectively, n = 636 and n = 606.

**Table 3.** Odds ratio analysis of symptom severity distribution according to each Jeopardy Index groups in Brazilian university students.

Anxiety symptoms	**OR**	**95% CI**	**p-value**
**Moderate**			
2	0.99	(0.54–1.82)	0.99
3	0.90	(0.51– 1.59)	0.73
4	0.95	(0.52– 1.74)	0.88
5	1.62	(0.80– 3.27)	0.17
**Severe**			
2	1.59	(0.78– 3.25)	0.20
3	1.42	(0.71– 2.80)	0.31
4	3.38	(1.77– 6.43)	**<0.01**
5	4.50	(2.12– 9.52)	**<0.01**
**Depressive symptoms**	**OR**	**95% CI**	**p**
**Moderate**			
2	1.23	(0.65–2.31)	0.51
3	1.95	(1.05–3.62)	**0.03**
4	1.46	(0.78–2.71)	0.22
5	1.92	(0.88–4.22)	0.10
**Moderate to severe**			
2	1.59	(0.79–3.21)	0.19
3	2.02	(1.00–4.08)	**0.05**
4	1.57	(0.77–3.18)	0.20
5	2.76	(1.19–6.37)	**0.01**
**Severe**			
2	1.14	(0.46–2.81)	0.77
3	2.19	(0.95–5.05)	0.06
4	3.19	(1.47–6.90)	**<0.01**
5	6.23	(2.59–14.95)	**<0.01**

Note: The references groups used for the analyses were the most privileged ones in the Jeopardy Index classification (0 and 1) and the mild symptoms severity.

## DISCUSSION

The prevalence of self-reported anxiety and depression symptoms was notably high in this study, 43.5% and 51%, respectively. Studies with university students show high prevalence of mental health problems^26^, with a heightened average level of depressive symptoms merely two months after entering college^27^, and a trend of worsening perception of mental health throughout the academic lifespan^26^. Given the potential negative impact of the academic journey on mental health, this study conducted adjusted odds ratio analyses for age and the enrollment in postgraduate programs, but no significant differences were observed with the non-adjusted analyses (Table 3).

Despite the already elevated prevalence of mental health problems in college students, it is crucial to underscore the additional negative impact of COVID-19 pandemic for the increase in symptoms of depression, anxiety, stress, and sleep problems in the young population^28^. In Brazil, the public universities have maintained stringent safety restrictions due to COVID-19 pandemic, with most classes remaining online until the first semester of 2022^29^.

Due to socioeconomic disparities and lack of technological infrastructure to promptly address the new demands imposed by the COVID-19 pandemic, several universities went through a prolonged period without even online classes. Considering the Brazilian social context, we highlight that this study was conducted during the initial year of transitioning back to normality with in-person classes, and this particular juncture could itself contribute to heightened anxiety levels among participants^30^.

Beyond the overarching socio-political context and the heightened prevalence rates in the general population, the Jeopardy Index approach showed that the less privileged cluster groups accumulate higher prevalence for anxiety and depressive symptoms. Concerning anxiety, 54.3% of individuals in group four and 63.1% in group five surpassed the anxiety scale cutoff point. Regarding depression, 59.6% of group four and 76.3% of group five scored above the cutoff point. The severity of symptoms showed a clear trend of escalation for these groups, illustrated in Figure 4, from mild to severe symptoms, corresponding to the group classification. Specifically, for severe anxiety symptoms, groups four and five exhibited similar higher prevalence rates (32.1% and 36.8%, respectively), with no statistical difference between them, but notably different from all other groups. Similarly, for depression, group five had the highest prevalence (39.4%), followed by group four (19.2%), with no significant statistical difference between these two groups.

Groups four and five, characterized by an accumulative oppressive characteristics, probably face a higher risk of enduring social stigma, injustice, or oppression throughout their lives^31^. These groups primarily consisted of women (81.3% and 99.4%), Black or Mixed-race individuals (87.2% and 94.7%), and those with lower incomes (87.1% and 97.9%). These shared characteristics delineate a social group historically subjected to various forms of structured oppression, including sexism and racism^23 , 31^. A meta-analysis, encompassing 37 studies and a total of 76,608 undergraduate and graduate students from 20 Low-and Middle-Income Countries (LMCs), revealed an overall prevalence of 24.4% for depressive symptoms, with a highlighted gender disparity regarding suicidal ideation (women = 20.7%; men=15.5) and significant risk ratio; RR = 1.9; p < 0.01)^32^.

The theory of intersectionality posits that systems of inequality, such as racism and sexism, interact in intricate ways, mutually reinforcing each other and giving rise to unique social contexts where privilege and oppression can coexist^12^. It suggests that a complex social landscape, comprising multiple disadvantaged identities in combination, results in what is termed multiplicative disadvantage. This concept implies that the effects of various identity combinations exacerbate one another, surpassing a mere cumulative or moderating impact^33^. Conversely, certain complex contexts, such as those inhabited by affluent, heterosexual, White men, are theorized to experience multiplicative advantage owing to the interconnected nature of intersectional theories^23^.

Thus, the emergence of mental health risks and the observed disparities in depressive symptom trajectories within and across various social groups are notably influenced by the diverse social realities experienced by individuals at different intersections of racial/ethnic and gender hierarchies. As depressive symptoms hinder access to social, economic, and political resources, disparities in mental health may perpetuate broader social inequality patterns^34^.

Despite the similarities, an exclusive characteristic of group five is that it comprises entirely non-heterosexual individuals. This demographic encompasses another historically marginalized social group that experiences various forms of violence, endured suffering, and engages in struggles for equal rights^35^. According to the Associação Nacional de Travestis e Transexuais (ANTRA – National Association of Travestis and Transexuals), Brazil has held the unfortunate distinction of being the country with the highest number of murders of trans individuals worldwide, for fifteen consecutive years^36^. Most of these victims (80%) were young, Black or Mixed-race trans individuals, openly embracing feminine gender identities, specifically as trans women^36^.

This alarming figure underscores the substantial violence that the LGBTQIA+ community faces daily in this country. It further supports studies indicating that non-heterosexual individuals are at a 1.5 times higher risk of encountering mental disorders, experiencing suicidal thoughts, and engaging in intentional self-harm compared to their heterosexual counterparts^37^. Increasing evidence gathered over the past few decades demonstrates that sexual minority youth and adults exhibit notably poorer mental health outcomes than their heterosexual peers^35 , 37^.

The minority stress theory highlights that stigma, discrimination, and victimization stemming from a homophobic and violent culture contribute to distinct stressors experienced by non-heterosexual individuals^35^. These stressors encompass regular discriminatory experiences, such as micro aggressions, along with the fear of rejection, self-devaluation due to internalized homophobia, hiding one’s identity, and other stigmatizing factors^37^. These pressures may intensify feelings of hopelessness and helplessness, ultimately contributing to the development of depression and suicidal thoughts^38^.

Despite the already elevated levels of depression and anxiety symptoms among university students, the Jeopardy Index approach revealed a specific subset of students at a significantly higher risk of experiencing severe symptoms. This group primarily consists of individuals from the LGBTQIA+ community, demonstrating nearly nine times higher odds for severe depressive symptoms and six times higher odds for severe anxiety. Additionally, the groups exhibiting higher prevalence and elevated risk rates predominantly comprised women of color and individuals with lower income levels.

A comprehensive study conducted in Brazil, using data from the National Survey of Health (PNS), highlighted the public health issue of under treatment in mental health, with more than 70% of depressed adults not receiving any care^39^. The study revealed that individuals with lower incomes were more likely to experience depression, while Black or Mixed-race individuals were more prone to untreated depression^39^. Another alarming statistic in Brazil is that suicide is the fourth leading cause of death among adolescents and young people, with higher rates observed among Black and Mixed-race individuals, who have a 45% higher risk of committing suicide^40^.

To identify the symptom severity of college students’ mental health problems is crucial, given the positive association with risk for suicidal thoughts and self-injurious behavior^41^. The Jeopardy Index approach used in this study represents an effort to integrate the perspectives of social sciences into quantitative research on mental health, bringing up relevant social factors that are known as risk factors for mental health issues. However, the study has some limitations that need to be emphasized.

First, the self-reported instruments to assess depression and anxiety symptoms could be misinterpreted by the students, and we could have a self-selection bias that inflates the prevalence of symptoms in the recruited sample. Second, it is crucial to underscore that the findings from a cross-sectional study offer valuable indications deserving attention, however, their interpretation should be approached with caution due to their inherent limitation of not testing cause and effect. Moreover, the multiple jeopardy theory of intersectionality is a complex phenomenon that cannot be reduced to an additive concept^23 , 24^. Nevertheless, identifying students in urgent need for mental health support is the initial step to provide necessary attention, prevention, or interventions to mitigate mental health challenges in the university settings.

The Jeopardy Index approach showed that severe symptoms of anxiety and depression appear concentrated within a specific social group. It was revealed that women of color, with lower income, and those identifying as non-heterosexual exhibited nearly nine times higher odds for severe depressive symptoms and six times higher odds for severe anxiety symptoms. Despite the high overall prevalence of mental health issues, individuals with those social oppressive characteristics are experiencing significantly greater distress. This underscores the urgent need to incorporate social determinants of mental health into the screening processes for mental health problems within university settings.
