# Erratum

**DOI:** 10.11606/s1518-8787.2025059006197err

**Published:** 2025-06-09

In the article “Intersectionality and mental health in university students: a jeopardy index approach”, DOI https://doi.org/10.11606/s1518-8787.2025059006197, published on theRevista de Saúde Pública. 2025;59:e3, RSP corrects:

**Authors (page 1):**

Where it reads:

Daniel Alves Pires

It should read:

Daniel Alvarez Pires

